# A NFAT decoy approach to inhibit cardiac hypertrophy

**DOI:** 10.1007/s00424-021-02637-9

**Published:** 2021-11-12

**Authors:** Joerg Heineke

**Affiliations:** 1 Department of Cardiovascular Physiology, European Center for Angioscience, Medical Faculty Mannheim, Heidelberg University, Ludolf-Krehl-Str. 7-11, 68167 Mannheim, Germany; 2 German Center for Cardiovascular Research (DZHK), partner site, Heidelberg/Mannheim, Germany

Chronic heart failure (CHF) is a very common disease that affects around 64.3 million people worldwide [2]. It develops mainly as consequence of ischemic heart disease and pathological pressure overload (e.g. aortic stenosis or arterial hypertension), but also due to volume overload, diabetes mellitus or genetic cardiomyopathy. Irrespective of the etiology of CHF, an important common feature of the failing myocardium is hypertrophy, which results from overgrowth of single cardiomyocytes [4]. In contrast to physiological hypertrophy (e.g. in postnatal heart growth, pregnancy or athletes), pathological hypertrophy is associated with disease, interstitial fibrosis, reversion to an embryonic gene expression pattern and is only partially reversible. It is triggered directly by mechanical force and by an increased local and systemic abundance of growth factors, such as angiotensin II, (nor-)epinephrine, aldosterone and others. Current therapeutic strategies almost exclusively target these neurohormonal factors, for example by the use of ACE inhibitors, angiotensin receptor antagonists, beta-receptor blockers, and aldosterone antagonists. Although these medications markedly improved the outcome of CHF, its morbidity and mortality remain high [2]. One possible reason is that pathological hypertrophy and myocardial fibrosis are only insufficiently targeted or reversed [3, 6].

**Fig. 1:**
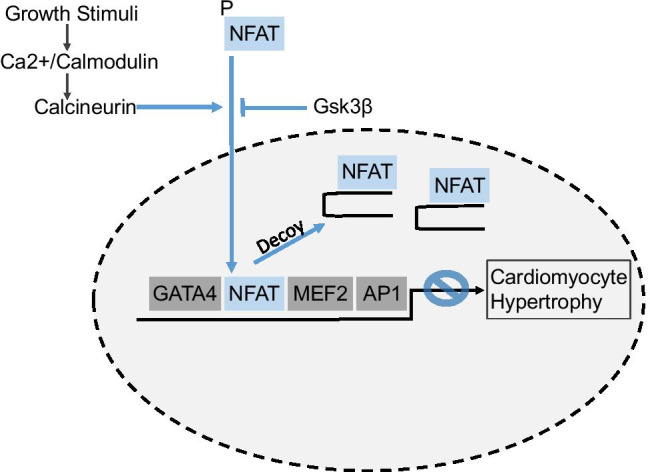
Growth stimuli trigger increased calcium levels in signaling domains in the cytosol, leading to calcium/calmodulin dependent activation of calcineurin in cardiomyocytes. Calcineurin dephosphorylates NFAT that translocates to the nucleus to induce pro-hypertrophic gene expresssion in concert with other transcription factors, like GATA4, MEF2 and AP1, which stabilize NFAT binding to DNA. NFAT nuclear translocation is antagonized by NFAT kinases, such as Gsk3β. NFAT decoy hairpins inhibit the binding of NFAT to DNA and thereby counteract cardiomyocyte hypertrophy

The binding of growth factors to their receptors activates an intricate web of intracellular signaling cascades that converge on effector kinases, transcription factors, and epigenetic regulators to induce cardiomyocyte hypertrophy [4]. Currently, however, no treatment is available to target any of these nodal intracellular regulators. Remes et al. have now tested a novel strategy to inhibit the transcription factor nuclear factor of activated T cells (NFAT) in order to reduce cardiac hypertrophy [7]. Four NFATc genes, *nfatc1-c4* were identified. They are all expressed in the mouse (and human) heart, although *nfatc2* is clearly the highest expressed form, followed with some distance by *nfatc3* [1, 8]. At their N-terminal end, NFATs exert a stretch of conserved phosphoserine residues, which are dephosphorylated by the calcium dependent phosphatase calcineurin [5]. NFAT dephosphorylation triggers its nuclear translocation and can be reversed by NFAT kinases (like glycogen synthase kinase 3ß, Gsk3β), enabling nuclear export. NFAT contains an imperfect Rel homology domain that only weakly binds DNA at the consensus sequence (A/TGGAAA). To strengthen NFAT/DNA interactions, NFATs interact cooperatively with other transcription factors such as AP-1, GATA4, and MEF2, leading to the integration of multiple signaling pathways [5]. Remarkably, although cardiac NFATs play a physiological role during heart development, in the adult mouse NFAT transcriptional activation was mainly observed during pathological hypertrophy (e.g. in pressure overload), but not physiological exercise [9]. Accordingly, genetic mouse models had shown that especially NFATc2 and NFATc3 are necessary for cardiac hypertrophy due to pressure overload, but not running exercise [1, 8]. Together this suggested that NFAT might be a good therapeutic target to selectively inhibit pathological hypertrophy. Remes et al. designed a decoy oligonucleotide (dON) approach to counteract NFATc1-c4 by mimicking the NFAT consensus-binding site and thereby scavenging cellular NFAT to prevent it from interacting with DNA sites on promoter and enhancer regions of target genes [7]. Indeed, this strategy did not affect nuclear localization of NFATs, but interfered with DNA binding and the induction of NFAT dependent genes, such as *rcan1*. Usually, transcription factor decoys are delivered as double stranded DNA in a sustained manner, but this is difficult in the heart without invasive methods and inefficient due to low DNA uptake by cardiomyocytes. Remes and colleagues therefore developed an approach using cardiotropic adeno-associated vector 9 (AAV9) to express an NFAT decoy RNA hairpin, requiring only one intravenous injection. When the AAV-NFAT-dON was delivered 2 weeks before pressure overload was induced by transverse aortic constriction (TAC), the authors observed less cardiac hypertrophy, less cardiac fibrosis and better preserved systolic heart function until 6 weeks after TAC. Importantly, the authors also tested a clinically more relevant scenario: They injected the AAV-NFAT-dON, 3 days *after* TAC, and still found an effectively improved heart function, reduction in hypertrophy and fibrosis similar as with prophylactic treatment (Fig. 1).

This study demonstrates how effective an AAV-based RNA hairpin transcription factor decoy can be in the treatment of cardiac hypertrophy and failure and opens up many new possibilities for targeted molecular therapies. Before these can become reality, a few important questions need to be solved and roadblocks removed. First, additional mouse studies are needed on the NFAT-decoy approach to test it for longer treatment durations, in additional cardiac diseases (for instance myocardial infarction, genetic cardiomyopathy) and under consideration of typical co-morbidities (e.g. diabetes) and older age as observed in patients. Furthermore, one needs to move to studies in larger animals, such as pigs, to test whether the NFAT decoy approach is still effective there. If that is the case, one could consider moving toward humans. Which patients would benefit from NFAT decoy treatment, and at which disease state could this approach be effective? Some clues to answer these questions could be derived from mouse and pig studies suggested above. One major hurdle is related to the AAV approach: sufficient AAV-based expression in the myocardium of patients is not yet achievable, and neutralizing pre-existing antibodies against the AAV capsid excludes a large group of patients from the start [10]. Capsid engineering and other innovative approaches are needed to remove this roadblock. When successful, NFAT decoy therapy could indeed become an effective weapon against pathological hypertrophy.
